# Zebrafish as a model for kidney function and disease

**DOI:** 10.1007/s00467-018-3921-7

**Published:** 2018-03-03

**Authors:** Priya Outtandy, Claire Russell, Robert Kleta, Detlef Bockenhauer

**Affiliations:** 1 Centre for Nephrology, Royal Free Hospital/Medical School, University College London, 1. Floor, Room 1.7007, Rowland Hill Street, London, NW3 2PF UK; 2 Department of Comparative Biomedical Sciences, Royal Veterinary College, Royal College Street, London, NW1 0TU UK

**Keywords:** Kidney, Zebrafish, Chronic kidney disease, Polycystic kidney disease, Acute kidney injury, Animal model, Renal diseases

## Abstract

Kidney disease is a global problem with around three million people diagnosed in the UK alone and the incidence is rising. Research is critical to develop better treatments. Animal models can help to better understand the pathophysiology behind the various kidney diseases and to screen for therapeutic compounds, but the use especially of mammalian models should be minimised in the interest of animal welfare. Zebrafish are increasingly used, as they are genetically tractable and have a basic renal anatomy comparable to mammalian kidneys with glomerular filtration and tubular filtration processing. Here, we discuss how zebrafish have advanced the study of nephrology and the mechanisms underlying kidney disease.

## Introduction

Kidney disease is a global problem with around three million people diagnosed in the UK alone and up to a million people undiagnosed [1]. Factors such as rising obesity, diabetes and hypertension have been shown to play a role in the manifestation of kidney dysfunction, but more research is needed to understand the pathophysiology behind the disease. Animal studies are a major tool in research, providing better understanding of normal physiology and mechanisms of disease and to test potential therapies. Zebrafish (*Danio rerio*) have been established as a model for development and reproduction studies since the 1930s [2]. But advances in technology have facilitated the growth of zebrafish as models to study organ-specific diseases, such as kidney disorders.

Zebrafish embryos at the earlier stages of development (before 5.0 days post-fertilisation) are considered to experience little to no pain and therefore are not protected under the animal experimentation regulations, thus making them a useful replacement model for mammalian models, such as mice and rats [3]. Zebrafish sperm can also be cryopreserved, reducing the need for live culture and therefore decreasing resource use [4].

Zebrafish has advantages over mammalian models such as mice (*Mus musculus*) in that pairings can produce a large number (~ 50–200) of offspring that can grow to adulthood within 3 months. This allows testing of various compounds and even drug screening is feasible and potentially high throughput. These embryos can be analysed for a phenotype of interest rapidly due to their transparent nature, and pigment can be reduced by chemical treatments and albino strains enabling phenotypic analysis at later stages [5]. They are especially useful for the study of genetic diseases, as they are very amenable to genetic manipulations, such as through microinjections of DNA and RNA even at the one-cell stage of life.

With regards to homology between the two species, there is high conservation of functional domains between human and zebrafish proteins, where at least 70% of zebrafish proteins have a human orthologue and 47% of human genes have a one-to-one association with orthologue in zebrafish [6]. Furthermore, genetic editing techniques such as transcription activator-like effector nucleases (TALENs), zinc finger nucleases and more recently, the Clustered regularly interspaced short palindromic repeat (CRISPR)-*Cas9* system can be easily employed in the zebrafish to study gene function and the consequences of its absence/modification.

Zebrafish have long been used in other fields of research to gain a better insight into various disease states. For example, via the use of chemicals, tumours can be induced in a number of organs proving to be useful models of cancer [7]. Zebrafish adults from the *c-myb*^*hyper*^ mutant line developed acute myeloid leukaemia-like or acute lymphoid leukaemia-like disorders due to the hyperactivity of the transcription factor c-myb (Myb proto-oncogene protein)—such abnormalities in man lead to many haematopoietic disorders [8]. Zebrafish have also been useful in the field of cardiology specifically in the study of heart regeneration mechanisms and can show cardiac failure when the same genes known to cause dilated cardiomyopathy in humans are knocked down in the zebrafish [9]. A significant amount of research has been carried out to study renal function and disease using zebrafish. Here, we will review kidney-related research in zebrafish and discuss the advantages and potential caveats involved of using this organism as a model.

## General aspects of the zebrafish model

Forward and reverse genetic approaches can easily be used in the zebrafish to enable the study of genes. Forward genetic approaches involve random mutagenesis in the embryo to alter gene function and assessing phenotypes as a starting point to find the causative gene [10]. Zebrafish screens are feasible and were first used to identify genes involved in vertebrate development [11].They have also shown to be successful in isolating candidate genes responsible for human diseases [12]. This is an unbiased approach in contrast to reverse genetics in which a specific gene of interest is disrupted to study the effects in the embryo.

A summary of the various genetic manipulations that can be used in the zebrafish is described here:

### Transient gene suppression: morpholinos

Another tool is the use of morpholinos which are targeted antisense oligonucleotides with modified bases that have commonly been used since the 1990s, to transiently knockdown a gene of interest in zebrafish [13]. They were first employed in the zebrafish in the 2000s and have the advantage of specifically blocking mRNA transcripts of interest with higher efficiency than other antisense oligos [14]. They are useful as a first approach to studying a particular gene or multiple genes in the absence of a genetic mutant [15].

Nasevicius and Ekker (2000) were able to show phenocopies for many known gene mutations such as the *one-eyed pinhead* confirming that morpholinos can successfully affect the majority of cells in the zebrafish [16]. It is essential that the optimum dose of morpholino is determined to avoid any pitfalls in mistaking the effect of toxicity as a gene-specific phenotype. Morpholinos can often exhibit phenotypes which are the result of off-target effects leading to uncertainty as to whether the phenotype is truly the result of a knockdown of the targeted gene. A common phenotype observed with morpholinos consisting of neural toxicity had been shown to be the result of activation of the p53 pathway [17]. These off-target effects that trigger the p53 pathway have been shown to be present in 15–20% of morpholino work injected at standard concentrations and can be problematic for work involving gene-specific activation of the p53 pathways [17]. Moreover, injections of p53 morpholinos with the gene-specific morpholino has become an important control step in distinguishing true phenotypes from off-target effects [18].

A recent paper showed that when CRISPR/*cas9-*induced mutants were examined alongside morpholino morphants, they exhibited a milder phenotype suggesting that there are wider off-target effects in the morpholino morphants that are contributing to the severe phenotype [19]. It has been suggested that milder phenotypes observed in mutants are thought to be the result of compensatory mechanisms where subsets of genes can be upregulated in response to loss of gene function [20]. This was not observed in morphants with morpholino use which can account for the more severe phenotypes but it could also be said that morpholinos can show the function of a gene more directly [15].The efficacy of the morpholinos is typically limited to around 3 days post injection [21]. Though morpholinos can be assessed for phenotypes quite rapidly they are transient, and it is more desirable to have a stable mutant to study long term.

There is also a growing requirement to validate morpholino work with genetic mutants so that phenotypes can be assessed for gene specificity as well as studying the effects long term. Determining the morpholino dose is also crucial and it is suggested that phenocopying known mutants as a reference is a suitable approach. Morpholinos should remain a valuable tool in zebrafish research providing that other guidelines are followed along with appropriate genetic mutant controls [16, 22].

## Forward genetic approaches

### Random mutagenesis

The most common method of random mutagenesis uses the methylating agent ethylnitrosourea (ENU) where exposure of the mutagen can create hundreds of mutations in male germ cells which are then grown to adulthood and crossed to wildtype females to create the F1 generation [23]. The F1 generation is outcrossed to obtain F2 generation carriers and the heterozygous carriers are then incrossed to get the F3 generation to study the homozygous portion of the progeny for phenotypes [23].

Point mutations are the most common outcome from this method [24]. Mutations with ENU have a high mutagenic rate so there is a higher germline transmission rate to future generations and the method offers a non-biased approach to identify genes that previously would have never been implicated with specific roles [25]. In zebrafish, the first ENU screen successfully identified over 600 genes with mutant phenotypes thought to be involved in embryonic development [11]. However, ENU mutagenesis requires positional cloning to be undertaken where the disease and candidate gene are colocalised onto a chromosomal region which is seen as more laboursome in comparison to other methods [26].

### Insertional mutagenesis

This method involves the addition of exogenous DNA, commonly pseudotyped retroviruses that can result in around 1 in 80 insertions disrupting gene function and causing a mutant phenotype [27, 28]. This forward genetic method is typically used to identify genes associated with a particular phenotype. This strategy is seen as a better alternative to ethylnitrosourea (ENU)-induced mutagenesis as the genetic sequence of the vector provides a distinctive identifier for cloning, making screening for mutants easier [29, 30].

## Reverse genetic approaches

### Targeting induced local lesions in genomes

One reverse genetic approach is targeting induced local lesions in genomes (TILLING) to study a specific gene and combines the use of chemical mutagenesis with polymerase chain reaction (PCR) to identify mismatches in mutant and DNA strands during annealing. At the mismatch site, a single strand nuclease, e.g. *CEL1* (purified from celery), cleaves the DNA which can be analysed with sequencing methods [31]. Adapting this approach in zebrafish was initially carried out by Fritz et al. who used a multiplex PCR to target a number of zebrafish genes to test on haploid offspring of female fish that were fertilised with gamma-radiated sperm [32]. The study was able to recover 15 mutants such as the *msxB (*muscle segment homeobox B) mutant which had no PCR product detected and displayed abnormal phenotypes such as a shortened body axis.

### Targeted germline mutations

Engineered endonucleases such as zinc finger nuclease and transcription activator-like effector nucleases (TALENS) also emerged as tools to create site-specific genome modification by introducing double-stranded breaks in a target region. Zinc finger nucleases (ZFN) are based on the theory of recognition of a few bases of sequence by each zinc finger and *Fokl* (*Flavobacterium okeanokoites*), an endonuclease, which becomes active when it dimerises, causing a double-stranded break in the DNA. These mutations using TALENS and zinc finger nucleases are introduced due to inefficient repair of the double-stranded breaks. TALENS also involves *Fokl* nucleases but the DNA-binding domain is larger, consisting of conserved repeats [33]. However, it is not easy to assemble zinc finger domains to bind to an extended stretch of nucleotides with high affinity and TALENS can be harder to express into a cell due to their larger size [34]. Thus, despite the recent advances in forward genetics, these approaches do have their limitations.

Clustered regularly interspaced short palindromic repeat (CRISPR) is a relatively new discovery in genetics, which has opened the field of gene editing. It had originally been shown in prokaryotes as a naturally occurring innate defence system against invading phages and plasmids where it produces a mature CRISPR RNA (crRNA) complex to recognise target DNA sequence in the virus and employs Cas 9 (CRISPR associated protein) enzymes to ‘cut’ the target site [35]. By inducing a double-stranded break in the sequence, it prevents proliferation of the foreign genetic elements [36]. Unlike morpholinos, which induce transient reductions in gene expression, CRISPR/Cas 9 results in germline mutations, which may result in more stable phenotypes. Like morpholinos, CRISPR/Cas9 can also have off-target effects, although there are tools/methods available to try and minimise these effects [37, 38]. CRISPR/Cas 9 has currently being implemented to create insertions and deletions (InDels) in the zebrafish genome successfully to create loss of function models of targeted genes. It is also possible to ‘knock-in’ a selected mutation, which is a promising approach to replicate and assess specific mutations seen in patients.

Discussing specific details of gene manipulation technologies is beyond the scope of this review and the interested reader is referred to some recent comprehensive reviews [39, 40] [41, 42].

With reverse genetic methods, targeting genes that have paralogues in the zebrafish, i.e. homologous duplicate pairs, can result in challenges in mapping the gene that causes the phenotype. Zebrafish belong to the teleost group where approximately 350 million years ago, a genome duplication event occurred during its early evolution and divergence from mammalian ancestors [43, 44]. The outcome of this is that these paralogues, which are closely related to each other in sequence, can have redundant functions [45]. Thus, there is often a 2:1 ratio of orthologues between zebrafish and humans [46]. This can be a challenge for researchers as considerations need to be taken when working with zebrafish genes that are duplicated to identify the most likely orthologue to the human gene of interest, which may involve more methods to analyse the data. For example, it may not be enough to analyse BLAST results of zebrafish and human data alone as one study found that it could not predict the most appropriate orthologues in the Msh homeobox 1 (*msx*) zebrafish family of genes: *msxa*, *msxb*, *msxc*, *msxd* and *msxe* with the human *MSX1* and *MSX2* genes [47]*.* Syntenic regions may provide more insight into their orthologous relationships [47]. Moreover, despite the evidence of homology, some zebrafish orthologues may not have the same function as the human gene. For example, heterozygous and homozygous mutations in the human granulin (*GRN*) gene have been linked to frontotemporal lobar degeneration and neuronal ceroid lipofuscinosis [48], respectively. However, when zebrafish orthologues of GRN, *grna* and *grnb*, were knocked out either separately or as a double knockout, the models showed no differences in regards to morphological and movement phenotypes [49].

Currently, the latest version of the zebrafish genome assembly is GRCz10, but the genome assembly is still undergoing improvements due to some sequencing gaps in the genome and errors in mapping. Occasionally, the new update can result in relocation of genomic regions. This can be of special relevance when a mutant line is obtained to study a specific gene of interest and changes to the genome assembly assign the retroviral insertion to a different gene than originally assumed. Gaps in the genome assembly can also lead to challenges in accurately mapping mutations from insertional mutagenesis.

## The zebrafish kidney and the mammalian kidney

Zebrafish is a freshwater fish and thus, a key function of the zebrafish kidney is to excrete water and maintain osmoregulation [50]. Yet, there is a close functional analogy between zebrafish and mammalian kidneys, which makes zebrafish an excellent model for renal research. Zebrafish can be a useful organism to study processes such as glomerular filtration and renal tubular clearance as there is conservation of this mechanism from fish to mammals [51].

**Fig. 1:**
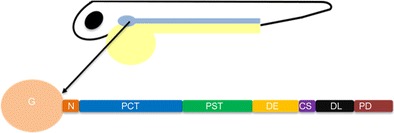
Schematic representation of the pronephros in the developing zebrafish. The image shows one pronephric tubule out of the bilateral organ consisting of the various segments. The distal early tubule is representative of the thick ascending limb, and the distal late segment is representative of the distal convoluted tubule in humans. The pronephric duct is analogous to the collecting duct in humans. The tubular epithelium then joins with the cloaca to complete the process. By the 28-somite stage, the pronephros is developed consisting of two parallel nephrons and glomerular filtration can commence from 2 days post-fertilisation. *G* glomerulus, *N* neck, *PCT* proximal convoluted tubule, *PST* proximal straight tubule, *DE* distal early tubule, *CS* corpuscles of stannius, *DL* distal late tubule, *PD* pronephric duct

Zebrafish have a pronephros and despite this being the most ‘basic kidney’ in vertebrate organisms, it has been shown to be comparable to the mammalian metanephros [52]. The zebrafish pronephros is comprised of two nephrons that derive from bilateral stripes of intermediate mesoderm, similarly to the mammalian kidney [53]. Two bilateral pronephric ducts are linked with glomeruli located in the midline of the embryo. The pronephric duct is lined by epithelium from the glomerulus to the cloaca, which is divided into segments comparable to the mammalian system, such as the proximal and the early distal tubule (Fig. 1) [52]. In addition, Wingert et al. found that many genes that are localised to specific segment in the mammalian kidney were also expressed in the pronephros. For example, solute transporters *slc9a3* and *slc4a4* are restricted to the proximal segments in both zebrafish and mammals and expression of *clcnk* which encodes a chloride channel in mammals in the distal regions are comparable to the *clck* expression in the distal part of the pronephros [51]. Like humans, zebrafish have a developed brush border membrane and also express megalin and cubulin receptors located in the proximal convoluted tubule segment to aid reabsorption of salts, sugars and small proteins [54]. While the zebrafish glomerular anatomy is comparable to the human glomerulus, another difference is that zebrafish have a mesonephric rather than metanephric adult kidney and have no bladder [53]. Moreover, the pronephros structure differs slightly in structure to the more specialised mammalian metanephros; for example, the pronephros does not have a thin limb segment between the proximal straight tubule and the thick ascending limb (equivalent to the zebrafish distal early segment).

## The developing kidney

There is also analogy in kidney development: zebrafish genes such as *pax2.1* (essential for kidney development in higher vertebrates) are also directing pronephric development resulting in the tubular segmentation discussed above [55]. In the intermediate mesoderm and then pronephric duct, the zebrafish protein Cadherin17, orthologous to human LI-cadherin (CDH17), is expressed to ensure the development of apical and basolateral membrane domains and for cell cohesion within the ducts [56]. The Notch signalling pathway has been shown to play a role in the developing pronephric nephrons as ligands such as *deltaC*, *jag1b and jag2a* are expressed and restricted to the proximal intermediate mesoderm [57].The pronephros localisation and segmentation is mediated by (*caudal*) *cdx* transcription factors which affect retinoic acid activity by increasing its production at the embryonic axis and then forming the pronephros at this site [51].

Zebrafish provided an insight into how nephrons differentiate into segments by studying the pattern of signalling pathways in both retinoic acid and *irx3b*. Morpholino knockdown of *irx3b* in zebrafish embryos showed lack of a mature distal segment as well as expansion of the proximal tubule boundaries and oedema at 72 h post-fertilisation indicating its involvement in demarcating nephron proximal and distal signals [58].Interestingly, zebrafish also have the ability to develop new nephrons (nephrogenesis) in response to injury in their lifespan in contrast to mammals [59]. By studying nephrogenesis in the zebrafish, researchers can test molecular pathways for roles in nephrogenesis, which can subsequently be further studied in murine models.

**Fig. 2:**
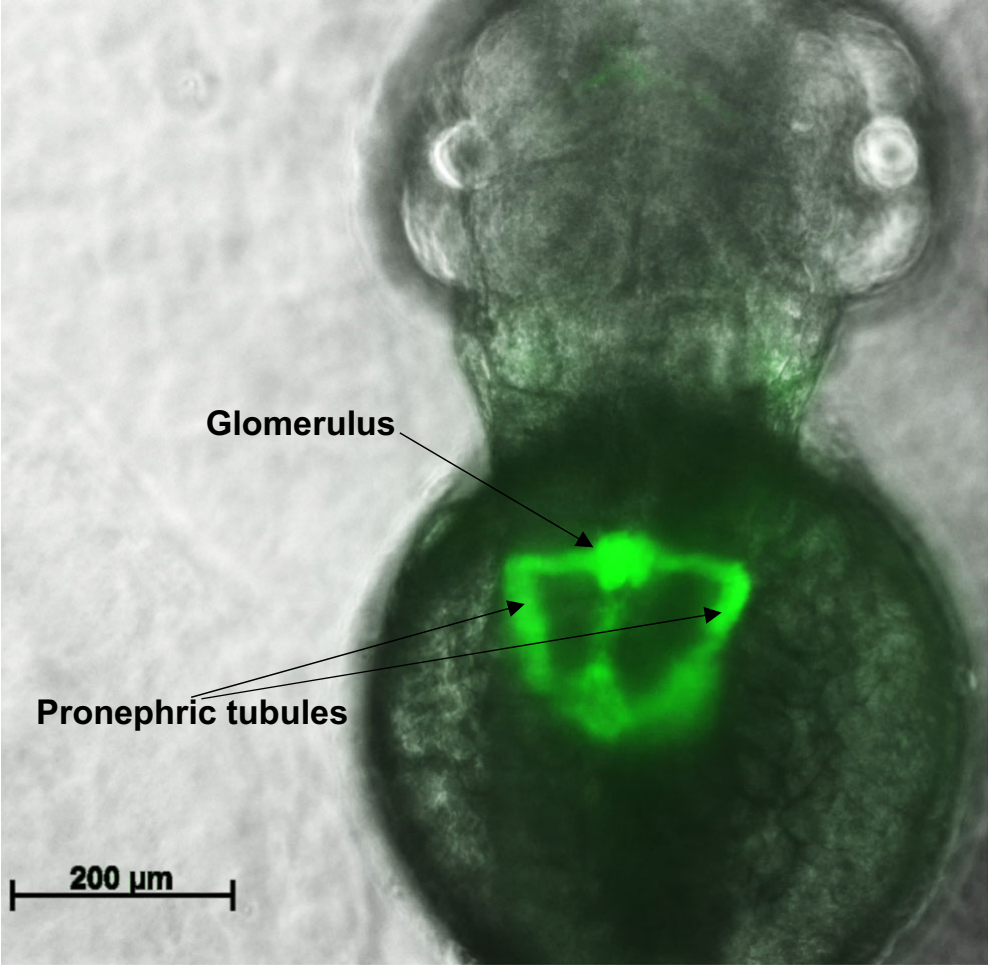
The developing kidney in a 48-h post-fertilisation zebrafish embryo. Shown is an image of a transgenic fish *Tg*(*wt1b:EGFP*), expressing the fluorescent protein E-GFP in kidney progenitor cells. The kidney is seen by its GFP expression, with notable structures labelled. The pronephric tubules have not yet reached their full length at this stage of development but will elongate further by 3.5 days post-fertilisation

Many zebrafish mutant lines exist today in biomedical research that are used to study kidney function and relate the findings to the human kidney. The *Tg*(*wt1b:EGFP*) zebrafish line [60], for example, enables the fluorescent visualisation of the proximal tubules and glomerulus via green fluorescent protein (GFP) (Fig. 2) and was used to show the importance of the Wilms tumour protein (*wt1*) in pronephros development, glomerular formation and nephrogenesis in zebrafish [60]. This is similar to its role in humans as *WT1* also plays a role in kidney development in mammals [61]. Mutations in *WT1* can lead to Wilms tumour, a paediatric kidney cancer [62].

## Functional assays to study kidney function

There are other tools available to study kidney function. Given the importance of the kidney for water excretion, the presence of oedema in a mutant fish can be an indicator of impaired kidney function. An inherent caveat of the zebrafish model is that it is often difficult to detect changes in the urine to assess the clearance of specific markers of interest as it is the level in the surrounding water rather than the pure urine that can be examined. Normal renal function tests routinely used in clinics are challenging to perform in zebrafish, due to small volumes of urine, and the fact that urine is released into the surrounding water. This can limit the utility of zebrafish as a model for specific tubular diseases.

Fluorescent assays have been useful in studying zebrafish models of kidney function and disease [63–65]. Typically, fluorescent tracers are injected in zebrafish embryos so that they can easily be tracked under a fluorescent microscope within an area of interest. Large molecular weight tracers are retained by the intact glomerular barrier and thus can be used to study glomerular function [54] [66, 67]. A transgenic line was developed to express vitamin D-binding protein tagged to green fluorescent protein (VDBP-GFP; combined size of approximately 87 kDa). When crossed with another transgenic line in which podocyte damage can be induced, the double-transgenic fish showed accumulation of VDBP-GFP within the proximal tubules indicative of a damaged glomerular filtration barrier (GFB) [68].

**Fig. 3:**
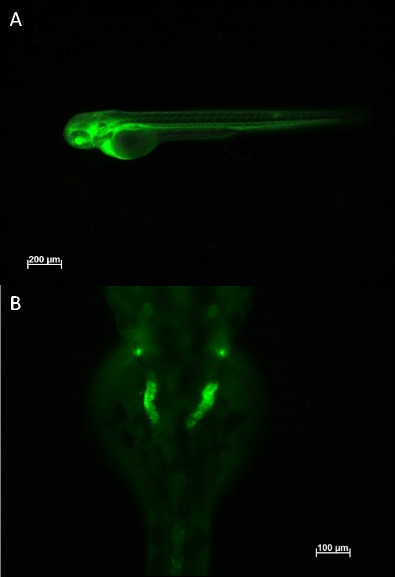
Injection of 10 kDa FITC-dextran into a wild-type zebrafish embryo at 72 h post-fertilisation visualised on a fluorescence inverted compound microscope. **a** Injection of the tracer into the common cardinal veins taken just after injection, showing uptake of the dextran into the blood vasculature of the embryo. **b** Dorsal visualisation of the dextran being taken up in to the pronephric duct and tubules after 20 h incubation post injection. Note that the tubules appear convoluted as they form a coiled loop around the glomerulus (not shown) and show reduced fluorescence in the rest of the embryo indicative of normal renal filtration processes

In addition, the disappearance rate of a low molecular weight fluorescent tracer can be used as a rough marker of glomerular filtrate rate. This technique can comprehensively be implemented using zebrafish embryos and the kidney visualised using a fluorescence microscope (Fig. 3). Impaired glomerular filtration will lead to retention of these dextrans in the fish, which can be visualised and measured. In our studies of the *kcnj10* morphant for example, we showed impaired renal clearance of a rhodamine-labelled 10 kDa dextran, when injected into the pericardium of the embryos. The loss of fluorescence from the fish correlates with the clearance of the dextran by the kidneys and loss of *kcnj10* in these morphants showed higher levels of fluorescence compared to controls [69].

Tracers can also be used to assess tubular function, specifically reuptake in the proximal tubule [70, 71]. Another important aspect of tubular function in zebrafish (a sweetwater fish) is excretion of water. Consequently, deletion of tubular transporters can lead to water retention, as we showed in the abovementioned kcnj10 morphants, which displayed a dilated pronephric duct and pericardial oedema [69].

## Zebrafish as a model of glomerular disease

Zebrafish have helped validate bioinformatic findings from genome-wide association studies (GWAS), which identify genomic regions that can then be investigated in the zebrafish. In one such study, two novel candidate proteins, SOS Ras/Rho guanine nucleotide exchange factor 2 (SOS2) and acid phosphatase 1 (ACPI), were predicted to be involved in early development and function of the kidney based on estimated glomerular filtration rate (eGFR), serum creatinine values and ‘variants genotyped’ from next generation sequencing. These patients were recruited from the ‘CKDGen consortium’ to assess the effect of rare variants on chronic kidney disease as the multiple common genetic risk variants could only explain a small proportion of variation with eGFR and urinary albumin-to-creatinine ratio (UACR) measures in these patients [72].When morpholinos against these genes were injected into zebrafish embryos, there were morphological changes in the renal tubule seen, e.g. elongation, and there was also reduced body length overall of the embryo [72]. To ensure that these changes were not a result of off-target effects, the morpholinos directed against the ‘ATG’ start site were validated with splice site morpholinos which also show similar phenotypes such as the reduced body length [72]. Embryos also displayed oedema at 72 h old and when assessed for glomerular function and tubular flow with injections of 70 kDa rhodamine-labelled dextran, these morphants showed decreased dextran clearance compared to controls [72]. This phenotype is also associated with the role of *sos2* and *acp1* in kidney development [72]. In humans, mutation with SOS2 is associated with Noonan syndrome which results in kidney dysfunction and though there is no current association between ACP1 and kidney function, it provides a useful target for further studies into kidney abnormalities.

Nephrotic syndrome has also been studied in zebrafish. Mutations in genes encoding proteins expressed in the slit diaphragm have been identified in patients with nephrotic syndrome, bringing the focus onto podocytes and their interactions during filtration [73, 74]. Mutations in the *phospholipase C epilson* (*plce1*) gene, whose normal function is required to initiate cell growth, differentiation and gene expression, were shown to cause early onset nephrotic syndrome and end-stage kidney disease [75]. Essential domains and residues in the *plce1* protein are highly conserved in zebrafish, enabling researchers to investigate the role of plce1 in podocyte function. Morpholinos against the gene orthologue were injected in zebrafish embryos to assess the journey of a 500-kDa tracer (FITC-dextran) perfused into the vasculature [75]. The study found that in control fish, there was retention of this large tracer in the glomerular vasculature and typical morphology. However, in *plce1* knockdown embryos, there was a breakdown in the glomerular barrier as expression of the tracer was visualised in the proximal tubule. Moreover, these embryos developed an oedematous phenotype with podocyte effacement classically representative of nephrotic syndromes.

A study of steroid-resistant nephrotic syndrome (SRNS) identified underlying mutations in the gene *FAT1* (FAT atypical cadherin 1) in some patients [76]. To understand the role of *FAT1* in glomerular function in vivo, the investigators knocked down *fat1* in zebrafish embryos from the transgenic line *Tg*(*wt1b:EGFP*) [76]*.* This transgenic fish line allows fluorescent visualisation of the glomerulus and proximal tubules [60].The study revealed that knockdown of *fat1* resulted in pronephric cyst development in morphants. The paper then investigated the link between fat1 and Rho GTPases. The Rho GTPases family include RAC1 (Ras-related C3 botulinum toxin) and CDC42 (Cell division control protein 42 homologue) which are both involved in cellular migration: CDC42 in particular is involved in filopodia formation and normal tubular function [77]. Knockdown of *fat1* in zebrafish caused a decrease in the amount of active RAC1 and CDC42 available and the addition of RAC1/CD42 was partially able to rescue the cyst phenotype observed [76]. The study showed that FAT1 is important for glomerular function as it is involved in cell migration of renal tubular cells and that activation of Rho GTPases can correct defective migration in the absence of FAT1 [76].

The gene *ELMO1* had been implicated in the development of diabetic nephropathy but its function in the nephron was relatively unknown. The zebrafish orthologue *elmo1* is expressed in the vasculature and in hyperglycaemic conditions, *elmo1* knockout mutants (via CRISPR/Cas9 system) were found to have an abnormal increase in the width of the pronephric system [78]. Moreover, they also exhibited a larger glomerulus and abnormal podocyte development. During filtration assays, the injection of 70 kDa dextran in the embryonic heart demonstrated abnormal filtration in the mutants with significant losses of fluorescence over time in comparison to CRISPR controls. This is thought to represent hyperfiltration as seen in early diabetic nephropathy [78]. The study was able to establish the protective effect of *elmo1* in diabetic nephropathy on kidney function and structure during hyperglycaemic conditions.

## Acute kidney injury

Zebrafish have also been used to study the effect of nephrotoxic agents such as cisplatin and gentamicin. Administration of these drugs induces oedema in embryos, as well as histological changes such as brush border flattening and debris in the tubular lumen [79]. Most importantly, injection of tracers such as dextran and inulin into these drug-treated zebrafish showed disturbances in the glomerular filtration rate as clearance is decreased relative to controls [79].

The E3-ubiquitin ligase, murine double-minute (*mdm2*) gene, regulates p53 induction and is a promoter of inflammation. It is also important for maintaining homeostasis of renal tubular epithelial cells (which normally divide at a slow rate) so that there is an adequate turnover of cells. Though *mdm2* inhibitors are being investigated as anti-cancer drugs [80], a recent study by Thomasova et al. showed that it also has a protective effect on renal tubular epithelial cells preventing acute kidney injury [81]. To test the importance of this gene in vivo, *mdm2* was knocked down using a morpholino approach in the *Tg*(*wt1b:EGFP*) transgenic line where apoptosis of renal epithelial cells measured by a terminal deoxynucleotidyl transferase (TUNEL (TdT)-mediated dUTP)) assay was observed as well as increased width of the pronephric tubules [81]. These morphants also exhibited pericardial oedema at 2 days old. Knockdown of *mdm2* using another transgenic line, *CADE* (*Tg* (*Casper*; *l-fabp:****D****BP-****E****GFP*), showed reduced fluorescence in the vessels indicative of a leaky filtration barrier [81]. The study highlights *mdm2* as an important gene for tubular epithelial homeostasis and survival.

## Tubular function

Despite the limitations of zebrafish in the study of tubular diseases, they have also been used to study disorders with tubular involvement. As mentioned previously, zebrafish morphants recapitulated the key features of EAST syndrome, representing the first zebrafish model of a tubulopathy [69]. More recently, zebrafish were used to study the role of *ocrl1* (associated with Lowe syndrome and Dent disease) [82]. The investigators were able to demonstrate the role of ocrl1 in proximal tubular function by showing impaired endocytosis within the pronephric tubules in *ocrl1*-deficient zebrafish embryos and thus established zebrafish as a useful model for the study of renal Fanconi syndromes. In another study, *ocrl1*-deficient zebrafish embryos also showed brain and eye reduction in which orcl1 is highly expressed, suggesting a developmental role for this gene within the central nervous system [83]. Previous work with zebrafish mutants has also provided evidence of Lowe syndrome as a ciliopathy where antisense morpholinos targeting the *ocrl1* gene showed cystogenesis and pronephros dilation, typically seen in other cilia-defective models [84]. Zebrafish were also successfully used as a model for nephropathic cystinosis, where patients exhibit renal Fanconi syndrome with progressive glomerular damage and multiple organ dysfunction [70]. Mutants homozygous for the *ctns* (cystinonin) gene showed cystine accumulation, delayed development and proximal tubular impairment with signs of glomerular damage recapitulating the patient phenotype [70].

## Polycystic kidney disease and other ciliopathies

A panel of zebrafish mutants with cystic kidney phenotypes identified 12 causative genes, which now constitute candidate genes for human cystic kidney disease identified by a forward genetic insertional screen [85]. Previously, the orthologues of two of the 12 genes, hepatocyte nuclear factor 1 (*HNF1*) and polycystic kidney disease 2 (*PKD2*), had been associated with human forms of polycystic kidney disease (PKD/PC) [86, 87], validating this panel approach. Mutations in the polycystins (PC), i.e. polycystic kidney disease 1 (*PKD1*) and *PKD2*, cause autosomal dominant polycystic kidney disease in humans [88]. It is thought that there is a ‘two-step’ model in the initiation cystogenesis consisting of germline mutations and somatic mutations in PKD in epithelial cells. This then results in proliferation of these cells and causes formation of cysts and tubular dilation.

Zebrafish embryos overexpressing the C-terminal polycystin fragment, by injection with exogenous mRNA or DNA, showed a marked ‘dorsalising’ effect where the tissue in the muscle and pronephric duct development were disrupted [89]. The study suggested that *polycystin-1* modulates the Wnt signal transduction pathway, cooperating together during vital stages of renal tubulogenesis [89]. Low et al. also showed that overexpression of *polycystin-1* (via injections of human *polycystin-1* mRNA encoding the PC1 tail into zebrafish embryos) resulted in cysts at 3 days post-fertilisation but with no ‘dorsalising’ effects. Though both studies show different phenotypes, they both implicate polycystin 1 as a modulator of the Wnt pathway, mainly inhibiting glycogen synthase kinase (GSK-3β) which normally phosphorylates beta-catenin for subsequent degradation [90, 91].However, the approach of Low et al. may better recapitulate the renal cyst formation seen in autosomal dominant polycystic kidney disease.

Morpholinos directed against *nphp1*, a gene associated with nephronophthisis, in zebrafish helped to corroborate the finding that mutations in this gene can contribute to the disease spectrum seen in Bardet-Biedl syndrome (BBS), a known ciliopathy [92]. Moreover, a zebrafish ciliopathy model of Meckel-Gruber syndrome (in which symptoms include renal cystic dysplasia) demonstrated the importance of the zebrafish paralogues *meckelin* (*mks*3) and *filamin A* (*flna*) in causing the disease. It was shown that *mks*3 localises to the primary cilia and *flna* is a binding partner that interacts at the apical cell surface [93]. When these genes were knocked down either separately or together in the zebrafish, phenotypes showed pronephric cyst formation along with notochord abnormalities suggesting these genes are also involved in Wnt signalling [93].

Zebrafish have also provided cross species conservation of a mutation in the Nek-family kinases, causing cyst formation in the connecting segments and cortical collecting ducts of mice [94]. Mutations in one family member, Nek8, are associated with polycystic kidney disease [94]. Using a morpholino approach in the zebrafish, embryos were injected with Nek8 antisense sequence resulting in cystic kidneys as early as 48 h post-fertilisation, supporting the phenotype seen in mice [94]. This data was able to provide evidence to support observations seen in vitro where mutated Nek8 showed defects in cytoskeletal structures in cell culture and loss of cell adhesion—this is thought to be a cause of cystic kidney disease.

## End-stage renal disease and apolipoprotein 1

Apolipoprotein 1 (*APOL1*) variants have been shown to increase the risk of progression onto developing end-stage renal disease (ESRD) [95]. These mutations are more prevalent in African populations compared to European American populations because of a protective effect against the African parasite *Trypanosoma brucei rhodesiense* infection. In order to study this further, zebrafish were used as they offer an advantage over mice models as there is no expression, nor orthologue, of APOL1 in the mouse and zApol1 is expressed in zebrafish podocytes. Olabisi et al. showed expression of these human APOL1 variants (G1 or G2) under the Gal4/UAS system in transgenic zebrafish resulted in glomeruli aberrations as shown by podocyte effacement and endothelial injury [96]. There was no tubular proteinuria detected via the renal excretion of GFP-vitamin D-binding protein where there is accumulation of the tracer within the proximal tubules, similar to the method employed by Zhou et al., but with puromycin aminonucleoside (PAN) as an inducer of podocyte injury. The study showed that the transgenic zebrafish expressing human APOL1 variants did not further potentiate the glomerular injury induced by PAN so though there were histological defects, it was not enough to cause renal dysfunction. The study suggested that presence of these variants may increase the onset of Apol1 nephropathy but a ‘second hit’ other than PAN may be required to observe kidney dysfunction [96]. In another study, knock down of the zebrafish orthologue, *zApoL1* in embryos resulted in oedema suggestive of a leaky filtration barrier [97]. This was confirmed with coinjection of 500 and 10 kDa dextran—in morphants, the pronephric tubules showed fluorescence from both markers after 24 h, whereas only the 10 kDa dextran was visible in the control pronephric tubules. This study showed the usefulness of zebrafish for the study of this gene. Unlike primates, in which there are up to five additional APOL genes, zebrafish have only one APOL orthologue eliminating the potential redundancy effect [97].

## Regeneration of the kidney

Zebrafish have also been used to study the possibility of regeneration after loss of function. It was shown that nephron progenitor cells can produce new nephrons in the adult zebrafish [59]. Moreover, using transgenic lines Tg (wt1b:GFP) and Tg(pod:NTR-mcherry) (the latter being a red fluorescent marker of glomerular podocytes), the investigators were able to track mesonephric nephrons from the moment of gentamicin-induced injury to regeneration of the mesonephros. They were able to show that expression of one of the *WT1* orthologues, *wt1b,* influences the development of new early progenitor cells proliferating into stage 1 nephron primordia by 4 days after injury in adult zebrafish [98]. When podocytes are injured via exposure to chemical agents, in this case MTZ (1-[2-hydroxyethyl]-2-methyl-5-nitroimidazole), *wt1b* showed expression in the Bowman’s capsule resulting in a podocyte-specific response and repair in the mesonephros [68]. The product of the other *WT1* orthologue, *wt1a*, also has a role along with FoxC1/2 transcription factors and notch signalling, to promote podocyte formation in zebrafish as morpholinos against these targets resulted in fewer podocytes developing.

## Conclusion

Various manipulations discussed here such as loss of function models and use of morpholinos can be implemented in the zebrafish to study disease states. We can study the impact of mutations in targeted genes that have a role in renal physiology by using zebrafish and use this knowledge to help understand more about the gene’s impact in humans, due to the genetic similarity. With targeted germline mutations, we can also assess the effect of specific mutations to assess the pathogenicity of genetic variants identified in patients with kidney disease. Zebrafish can also be used to study the basic renal physiology. Overall, zebrafish represent a useful model to study renal function and disease.
